# The Effect of Dioptric Blur on Sight-Reading Music

**DOI:** 10.1007/s44402-026-00045-z

**Published:** 2026-02-25

**Authors:** Deyue Yu, Melanie Akau, Susana T. L. Chung

**Affiliations:** 1 College of Optometry, Ohio State University, Columbus, Ohio USA; 2 School of Optometry, University of California, Berkeley, California USA

**Keywords:** Psychophysics, Reading, Visual acuity, Visual performance

## Abstract

**Purpose:**

Previous studies showed that dioptric blur degrades visual acuity and the critical print size for text reading. This study investigated whether dioptric blur affects sight-reading music, hypothesising that its effect on pitch and rhythm accuracy might differ.

**Methods:**

Eighteen young piano-players with normal vision played a Yamaha keyboard using the right hand while sight-reading short music pieces. Testing was conducted monocularly with dioptric blur (no lens, +1.00D, +2.00D, +3.00D and +4.00D) using convex trial lenses placed on the dilated right eye and with a 3 mm artificial pupil. Stimuli were 12-measure pieces (4 measures per line) with similar spatial complexity, printed in 11 note sizes ranging from –0.40 to 0.80 logMAR (defined as notehead height). A function of accuracy versus note size was obtained at each blur level. The threshold note size (TNS), defined as the note size corresponding to 80% maximum accuracy, was derived for each blur level for pitch and rhythm.

**Results:**

Visual acuity, pitch perception and rhythm perception were all susceptible to dioptric blur; however, this degrading effect can be compensated for by magnification. TNS was higher for pitch than rhythm across blur levels and remained unaffected until surpassing the critical blur level (CBL). Beyond the CBL (+1.49 D for pitch and +1.00 D for rhythm), TNS increased with blur, rising faster for pitch (0.19 logMAR per blur level) than rhythm (0.14 logMAR per blur level). The variation of TNS can be predicted partially by the change of visual acuity with increasing dioptric blur (*r* = 0.68 for pitch, 0.60 for rhythm).

**Conclusions:**

These findings highlight the importance of visual perception in music reading, with implications for music education and performance, particularly for individuals with visual impairments or in situations where visual clarity is compromised.

Key Points

- Dioptric blur negatively affects visual acuity, pitch perception and rhythm perception, but magnification can help counteract this degradation.
- Performance remained stable before reaching the critical blur level, after which increasing blur caused a sharper decline in pitch perception than in rhythm.
- The decline in performance for both pitch and rhythm can be partially explained by the corresponding decrease in visual acuity.

## Introduction

Music sight-reading, the ability to perform a musical composition directly from written notation without any prior rehearsal, is a complex skill that demands coordination of perceptual, kinaesthetic and cognitive processes [1–3]. Much like text reading comprehension in language arts literacy, music sight-reading is a fundamental component of music literacy [4]. It involves quickly identifying pitches, rhythms and other essential notation elements, and translating them into accurate sound in real time. The visual presentation of the music plays a critical role in this process.

The process of reading music may be analogous to reading written language, given that both rely on the interpretation of structured, rule-governed symbolic systems [4]. The left-to-right orientation of music notation mirrors that of written text. Just as text reading involves decoding letters and words to construct meaning, music reading entails decoding notes, rhythms and dynamics to interpret musical expression. Fluent comprehension in both domains requires efficient and accurate visual scanning. Notably, research has shown that performance on text reading comprehension is a significant predictor of music sight-reading ability [4].

Just as different fonts can affect text readability [5], the visual representation of the music, which varies among publishers, can influence its readability. Visual crowding has been suggested as a bottleneck on reading speed [6]; however, reading only slowed down when letter spacings [7, 8] or vertical word spacings [9, 10] were smaller than the ‘standard’ values in printed text. Similarly, in music reading, research has demonstrated that reduced visual crowding is associated with better reading performance [11, 12]. The strategic insertion of white spaces between musical fragments, presumably to reduce crowding, decreases sight-reading errors and enhances fluency, especially in visually congested passages [13]. In addition to crowding, research has demonstrated that dioptric blur degrades visual acuity and increases the critical print size required for text reading [14]. This raises the question of whether a similar effect of dioptric blur can be observed in music sight-reading performance. Exploring this question may provide valuable insights into the visual processing requirements associated with this intricate task.

A music note comprises two fundamental dimensions: the spatial dimension, characterised by pitch (indicating how high or low the sound is) and the temporal dimension, defined by rhythm (pertaining to the timing and duration of the sound in relation to a consistent beat). The placement of a note on the staff conveys information on pitch, while its value reveals information on rhythm. Although the horizontal position of notes can also provide cues about rhythm, this spatial encoding is not fully reliable and can be influenced by factors such as the density of notes within a measure. This study investigated the impact of dioptric blur on music performance by measuring pitch and rhythm accuracy as a function of note size across varying levels of dioptric blur. Pitch accuracy measures the degree to which performed notes match the intended frequencies, whereas rhythm accuracy assesses the alignment of note durations with the notated rhythm. These metrics collectively offer a quantitative evaluation of the accuracy in music performance. Given that different elements of printed music convey pitch and rhythm information, it was hypothesised that the effect of dioptric blur on the accuracy of pitch and rhythm might differ.

## Methods

### Observers

Eighteen young adults with normal vision (15 females and 3 males) with 5 to 19 years of piano playing experience took part in the experiment. None of the participants was a professional pianist. Informed consent was obtained from each observer prior to testing. The study was approved by the institutional review board at the University of California, Berkeley and conducted in accordance with the Declaration of Helsinki.

### Stimuli, Apparatus and Procedures

**Fig. 1:**
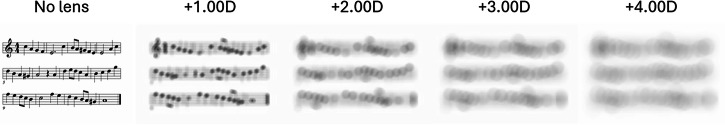
Simulated examples of music stimuli at the five levels of blur.

Eighty 12-measure pieces of music (four measures per line) with similar difficulty, number of notes and spatial complexity (see Fig. 1 for example) were composed by a coauthor with 16 years of formal music study, a bachelor’s degree in music performance and professional experience as a performing musician. The compositions were created specifically for this study, following consistent structural and stylistic guidelines to ensure uniformity across the pieces. These guidelines included maintaining a balanced melodic contour, consistent rhythmic patterns and avoiding advanced techniques beyond a beginner’s level. Specifically, the compositions were written in 2/4, 3/4 or 4/4 time signatures and the most complex rhythm used was the eighth note. Key signatures contained a maximum of two accidentals, limited to B‑flat major, F major, C major, G major and D major. Fifty of the 80 compositions remained within a maximum range of one octave, while the remaining 30 pieces featured melodic ranges expanding above an octave but not exceeding a perfect twelfth. Ledger lines were used in 23 of the 80 compositions, ranging from one to six ledger lines per composition. All pieces were typeset using Finale PrintMusic 2010 (MakeMusic, Inc.; finalemusic.com), which applied standard engraving defaults for horizontal spacing (i.e., spacing determined automatically by factors such as note duration and measure density) to ensure consistent visual presentation across stimuli. The number of notes per line was 12.2 ± 1.4, 13.3 ± 1.6 and 12.3 ± 1.4 for the first, second and third lines, respectively, with a total of 37.7 ± 3.9 (SD) notes per piece. The spatial complexity score (printed ink area) was computed for each music piece and each line of the piece. The coefficient of variation was then calculated to evaluate the relative variability of spatial complexity. It was confirmed that the spatial complexity was similar across music pieces and that the variability of spatial complexity was similar across the three lines of each music piece. The coefficients of variation were 3.24%, 4.08%, 3.84% and 2.53% for the first line, second line, third line and the whole piece, respectively. The stimuli were printed on white paper in high contrast (black on white), with one piece per page. Eleven note sizes ranging from –0.40 to 0.80 logMAR (defined as the height of a notehead or the interline spacing of staff) in steps of 0.10 logMAR were used.

Observers had their pupils dilated and cyclopleged using 0.5% proparacaine and 1.0% tropicamide, and viewed stimuli monocularly (right eye only) through a 3 mm artificial pupil mounted on a trial frame (an occluding lens was placed in front of the left eye). Five blur conditions were induced with convex trial lenses (no lens, +1.00D, +2.00D, +3.00D and +4.00D). Observers with refractive errors were fitted with trial lenses corresponding to their individual prescriptions. Additional power was also added to compensate for the test distance. Figure 1 shows simulated examples of how the music stimuli might appear at the five levels of spherical blur [14–16]. One measurement of visual acuity was obtained at each blur level using the Freiburg Vision Test (FrACT Version 3.6 by Michael Bach; michaelbach.de/fract/), employing Landolt C optotypes with a four-alternative forced choice paradigm and the build-in “Best PEST (Parameter Estimation by Sequential Testing)” procedure. Two viewing distances were used during visual acuity testing. The no‑lens and +1.00 D conditions were administered at 200 cm, whereas the +3.00 D and +4.00 D conditions were administered at 50 cm. The +2.00 D condition was tested at either 50 cm or 200 cm due to variability in setup. All reported acuity values were appropriately converted to account for differences in viewing distance. For each blur condition, five consecutive note sizes were selected based on the corresponding visual acuity. A piece of music at the smallest note size in the set was introduced for observers to practice. A Yamaha keyboard (Model number: PSR-E223; Yamaha Corporation; yamaha.com) was used. Its built-in metronome allows users to set the tempo by tapping a series of regular beats, enabling each observer to establish a consistent, self-selected baseline tempo for practice. These practice data were not included in the analysis. Two pieces of music were then tested at each note size. As data collection progressed, it became evident that adjustments on the range of note sizes were occasionally necessary to capture performance plateaus and thresholds more accurately. In such instances, no more than two additional note sizes were incorporated to encompass the limits of performance better. The order of testing across the five levels of blur and the five initially selected note sizes within each blur level was randomised. Additional note sizes could only be identified after testing the initial five sizes, and were administered immediately thereafter. None of the music pieces were used more than once for each observer. Since prior research has demonstrated that handedness does not affect piano performance [17], observers, regardless of their handedness, played the keyboard using only the right hand while sight-reading music pieces at a viewing distance of 60 cm. Responses were recorded using the hyperscribe function in the Finale PrintMusic 2010 notation program (MakeMusic, Inc.; finalemusic.com) and were scored offline for accuracy of pitch (spatial component) and rhythm (temporal component) afterwards.

### Data Analysis

The accuracy of pitch and rhythm was determined by calculating the proportion of correctly performed elements relative to the total number of expected elements. Accuracies were analysed subsequently using nonlinear mixed-effects models (NLME) [18–20] to assess the impact of dioptric blur. NLME provides robust model fit across observers, which was not achievable through individual-level fitting alone.

Statistical model fitting was carried out in Matlab utilising the Statistics and Machine Learning Toolbox (version R2023b; The MathWorks, mathworks.com). Logistic growth function

$$f\left(x,\varphi \right)=\frac{{\varphi }_{1}}{1+exp (-exp ({\varphi }_{2})(x-{\varphi }_{3}))}$$

was used to model the change of performance accuracy as a function of note size, where $x$ denotes note size in logMAR, ${{{{\boldsymbol{\varphi }}}}}_{{{{\boldsymbol{1}}}}}$ represents the maximum accuracy, ${{{{\boldsymbol{\varphi }}}}}_{{{{\boldsymbol{2}}}}}$ signifies the rate of change in accuracy with note size and ${{{{\boldsymbol{\varphi }}}}}_{{{{\boldsymbol{3}}}}}$ corresponds to the note size at which half of the maximum accuracy is achieved. NLME incorporates both fixed and random effects. Fixed effects, representing population parameters, are assumed to remain constant across data collection instances. In contrast, random effects are contingent upon the sample and function as additional error terms. In this analysis, blur level was included as a fixed effect and observers as a random effect. Separate analyses were performed for pitch and rhythm during the model fitting process.

## Results

**Fig. 2:**
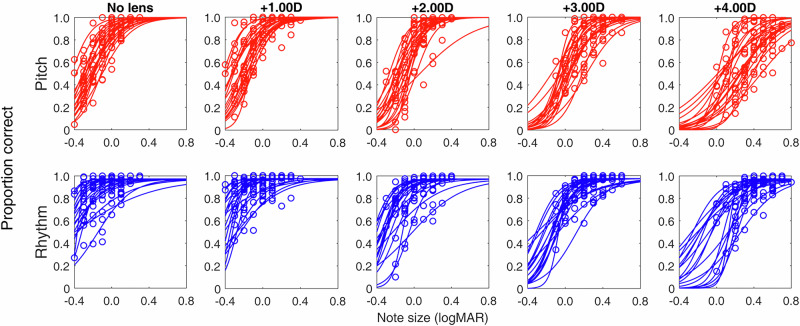
Accuracy of pitch (top row) and rhythm (bottom row) measured by the proportion correct is plotted against note size (logMAR) across five levels of blur: no lens, +1.00D, +2.00D, +3.00D and +4.00D. Each panel displays data from 18 individual observers. The curves shown in the data sets represent the best-fit functions, deriving using a nonlinear mixed-effects regression model.

**Fig. 3:**
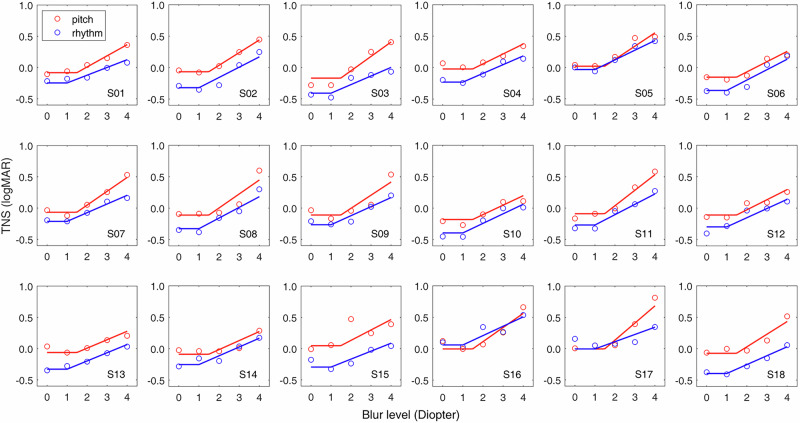
Threshold Note Size (TNS) as a function of blur level for pitch and rhythm. Each circle represents a data point from an individual observer.

At each blur level, the accuracy of both pitch and rhythm exhibited a monotonic increase with note size (Fig. 2). The threshold note size (TNS), defined as the note size corresponding with 80% maximum accuracy, was derived for each level of blur and for both pitch and rhythm. Across all blur levels, the TNS was smaller for rhythm than for pitch (*F*(1,17) = 70.05, *p* < 0.0005; Fig. 3). The average TNS values for pitch were −0.06, −0.08, 0.03, 0.19 and 0.43 logMAR for the five levels of blur (no lens, +1.00D, +2.00D, +3.00D and +4.00D), respectively. In contrast, the corresponding TNS values for rhythm were −0.24, −0.26, −0.12, 0.04 and 0.18 logMAR. There was no significant interaction between blur level and type of performance measure (*F*(4,68) = 2.09, *p* = 0.09), indicating that the impact of dioptric blur on TNS was similar for pitch and rhythm.

**Fig. 4:**
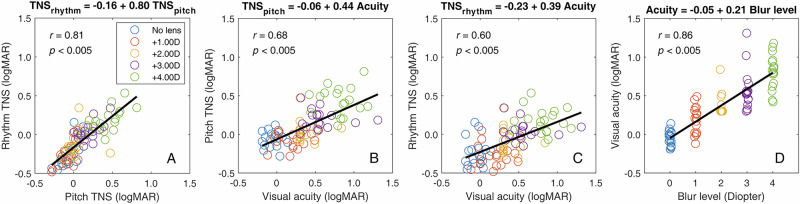
Scatter plot showing the relationship between **A** rhythm threshold note size (TNS) and pitch TNS, **B** pitch TNS and visual acuity, **C** rhythm TNS and visual acuity and **D** visual acuity and level of blur. Each panel contains data from five levels of blur and 18 observers. Each data point represents a measurement from a single observer at a specific blur level. A straight line is used to fit the data in each panel. In panel (**D**), a random horizontal jitter is added to each data point in order to separate overlapping points.

A bilinear function was applied to model the relationship between TNS and blur level, and to make further comparisons between pitch and rhythm. The bilinear function has been used previously to determine the critical value of an independent parameter that influences reading performance [9]. The parameter of interest in this study was the critical blur level (CBL), which was defined as the blur threshold below which TNS equalled that of the no-blur condition. During the curve fitting process, the slope of the first line was constrained to zero. The only variable parameters in the bilinear function are the minimum TNS, CBL and the slope of the second line. NLME was employed to analyse concurrently both population-level patterns and individual variations. As shown in Fig. 3, a similar trend was observed for pitch and rhythm. The TNS for both pitch and rhythm remained unaffected by the blur level until the CBL was surpassed. Beyond the CBL, TNS began to rise with increasing blur. Notably, the initial TNS or the TNS under the no blur condition was higher for pitch (-0.07 logMAR) compared with rhythm (-0.25 logMAR; *t*(17) = 7.54, *p* < 0.005). The CBL estimates were +1.49 D for pitch and +1.00 D for rhythm, with minimal individual variation observed. This minimal variation indicates that the population-level estimate can adequately represent individual observers, necessitating minimal adjustments for each observer. The slope of the second line was steeper for pitch (0.19 logMAR per blur level) than for rhythm (0.14 logMAR per blur level; *t*(17) = 5.14, *p* < 0.005), suggesting a more rapid increase in TNS for pitch with increasing blur. While this observation implies that dioptric blur affects pitch and rhythm performance differently beyond the CBLs, the magnitude of this difference is relatively small. Across blur levels, there is a strong correlation between pitch and rhythm TNSs (*r* = 0.81, *p* < 0.005; Fig. 4A).

As shown in Fig. 4B, C, significant correlations were identified between visual acuity and pitch TNS (*r* = 0.68, *p* < 0.005), as well as between visual acuity and rhythm TNS (*r* = 0.60, *p* < 0.005), indicating that visual acuity serves as a strong predictor of TNS for both pitch and rhythm. Figure 4D shows that the degree of blur was significantly correlated with visual acuity, accounting for 74% of the variation in visual acuity. The black line depicts the best-fit line across all five levels of blur. For each dioptre increase in blur level, visual acuity decreased by 0.21 logMAR. Upon post-hoc inspection of the data, it was identified that during the visual acuity assessment, for some observers, the +2.00D blur condition was implemented at a viewing distance shorter than appropriate for that blur level, likely inducing a ceiling effect. Observers affected by this might have shown better visual acuity had they been tested with a longer viewing distance. Therefore, the data were reanalysed, excluding the +2.00D blur level, to assess the impact on the results. The linear equation that described the relationship between visual acuity and blur level remained unchanged: visual acuity = −0.05 + 0.21 × blur level. The coefficient of determination, r^2^, had a slight increase, from 74% to 76%. Similarly, the linear functions that characterised the relationship between visual acuity, pitch and rhythm TNS also underwent minimal changes, with the r^2^ value rising from 46% to 51% for pitch and from 36% to 42% for rhythm. This suggests that the observed relationships are likely not driven by a single blur condition and that the predictive role of visual acuity in determining TNS holds across a range of blur levels. It also reinforces the validity of the linear relationship between dioptric blur and visual acuity, even in the presence of minor procedural deviation.

## Discussion

Music sight-reading is a crucial skill for musicians, enabling them to perform unfamiliar material accurately and fluently with minimal preparation. This ability involves rapid interpretation of musical notation, including elements such as pitch and rhythm and their subsequent translation into physical actions on an instrument [2]. This study aimed to explore how visual degradation, specifically dioptric blur, affected sight-reading performance. Given that different components of printed music convey information about pitch and rhythm, it was hypothesised that dioptric blur might affect the accuracy of pitch and rhythm differently. Pitch and rhythm accuracy were measured as a function of note size across varying levels of dioptric blur. Consistent with findings in text reading [14], the present results indicated that both visual acuity and the perceptual processing of musical notation are vulnerable to the degrading effects of dioptric blur. However, this degradation in pitch and rhythm perception can be compensated for by magnification (i.e., using larger note sizes).

Observers demonstrated a more accurate execution of rhythmic elements compared with pitch-related aspects of a musical piece. Across all levels of blur, achieving 80% maximum accuracy consistently required smaller note sizes (i.e., smaller TNS) for rhythm than for pitch. The mean difference in TNS between rhythm and pitch was 0.18 logMAR for the no blur condition and 0.18, 0.15, 0.16 and 0.25 logMAR for the four blur levels (+1.00D, +2.00D, +3.00D and +4.00D), respectively. A few factors may contribute to the higher accuracy in rhythm compared with pitch. First, the music pieces in this study were designed to incorporate only simple, easily recognisable rhythmic patterns. Additionally, the visual representation of rhythm, characterised by distinct notehead styles and stem patterns, provides a more intuitive and accessible means for observers to interpret and execute rhythmic elements. Consequently, recognising and executing rhythm may be more straightforward than performing pitch-related tasks, irrespective of the blur level. Second, pitch recognition requires precise localisation of specific notes on the staff. At high blur levels, the thin staff lines are among the first elements to be obscured in the blurred image (Fig. 1), complicating pitch identification. In contrast, even though the thin stem of the note may also be susceptible to blur, the beam connecting two eighth notes and the empty space or distance between notes carry redundant information regarding the duration of the note, facilitating a more robust interpretation of rhythmic patterns.

TNS remained unaffected by the blur level until a specific threshold (CBL) was surpassed. The CBL was higher for pitch compared with rhythm—TNS could tolerate up to +1.00D of blur for rhythm and +1.49D for pitch. The observed half-dioptre difference in blur tolerance between rhythm and pitch suggests that visual perception of rhythm-related elements exhibits greater sensitivity during the initial occurrence of dioptric blur compared with pitch-related elements. This may be because the clarity of note components conveying rhythm information (such as the stem of notes, the dot in a dotted half note and the fill of noteheads) is more susceptible to low levels of dioptric blur, whereas the relative positioning of notes that convey pitch information demonstrates better resilience to these blur levels.

Beyond CBL, pitch and rhythm performance begin to deteriorate as the level of visual blur increases. TNS increased with blur from an average of −0.06 to 0.43 logMAR for pitch and −0.24 to 0.18 logMAR for rhythm as blur level increased from zero to +4.00D. The steeper slope observed for pitch (0.19 logMAR per blur level) indicates that as visual blur intensifies, the ability to identify pitch accurately declines more rapidly than the ability to discern rhythm (0.14 logMAR per blur level). The variation of TNS can be explained partially by the change in visual acuity, which appears to have a greater influence on pitch perception than rhythm perception. In other words, pitch perception may rely more heavily on visual acuity than rhythm perception.

In interpreting these findings, several methodological constraints should be considered. First, the use of cycloplegia, an artificial pupil, trial frame and monocular viewing established a highly controlled optical environment but reduced ecological validity when compared with natural sight-reading conditions. Second, since the observers required different refractive corrections, the resulting peripheral blur profile was not consistent across individuals. Using contact lenses could have minimised the differences in off‑axis optical quality. Third, the restriction to playing with only one hand was implemented to reduce variability in motor coordination. Nonetheless, this simplification limits the generalisability of the results to typical two-handed performance. Fourth, although including observers with a broad range of piano experience increased the representativeness of the sample and enabled adequate sample size, this heterogeneity introduced large variability in sight‑reading strategies and performance levels, which might have contributed to greater variance in the pattern of findings. Another limitation is the use of composed musical pieces, which inherently include melodic and rhythmic structure. Because the notes follow musical relationships, observers may have relied on musical expectations rather than solely visual decoding. Although expectancy‑based processing is a natural part of real‑world sight‑reading, it limits the ability to isolate visual processing. Future studies could address this by incorporating stimuli with randomised pitch sequences, rhythms or pitch–rhythm pairings to help dissociate visual decoding demands from the influence of musical structure and expectation. Lastly, baseline pitch and rhythmic accuracies reflecting best attainable performance without visual restriction cannot be unequivocally determined from the present data. In the experiment, note sizes were individually selected for each observer and blur level to encompass a performance range suitable for estimating TNS, rather than to ensure asymptotic performance. As a result, the largest note size was deliberately positioned near, but not necessarily at, the performance plateau, and ceiling‑level pitch and rhythmic accuracies were not guaranteed even in the least visually demanding condition (i.e., the no-blur, largest-note-size condition). This design choice prioritised sensitivity to performance changes across note sizes over establishing a measure of optimal pitch and rhythmic accuracy.

The complexity of real-world musical compositions far exceeds that of the simple structures used in this study. More intricate melodic lines, harmonies and rhythmic patterns, coupled with increased visual crowding [21], may elevate sensory and cognitive demands significantly, thereby amplifying the challenges associated with both pitch and rhythm execution. Depending on the specific compositions, a more intricate musical piece may either exacerbate or mitigate the disparity between pitch and rhythmic accuracy.

The experience of the players may also play a significant role. It is possible that more experienced players make less errors overall and have smaller disparity between pitch and rhythm performance. None of the observers in the present study were professional players. While the range of piano playing experiences were recorded for these observers (5–19 years of piano playing experience), individual data were not obtained to perform correlation analysis. Future studies could benefit from collecting more detailed information on the musical backgrounds, including years of training, practice habits and proficiency levels. These data could provide valuable insights into how experience and expertise impact the effect of dioptric blur on music performance and the relationship between pitch and rhythmic accuracy.

Dioptric blur negatively impacts the perception of pitch and rhythm, with rhythm perception exhibiting greater sensitivity during the initial onset of dioptric blur and pitch perception deteriorating more rapidly as the blur intensifies. Notably, magnification can mitigate this detrimental effect of dioptric blur on pitch and rhythm perception. The strong correlation between visual acuity and TNS underscores the importance of visual perception in music reading abilities. These findings may have implications for music performance and education, particularly for individuals with visual impairments or in situations where visual clarity is compromised. For instance, musicians performing under suboptimal visual conditions such as low lighting, increased viewing distance, uncorrected refractive error or aging‑related visual decline may experience measurable decrements in sight‑reading accuracy. For music educators, the results highlight the importance of ensuring adequate visual clarity for beginning students, who may be especially vulnerable to visual limitations when learning to decode musical notation. Reduced clarity may increase cognitive load and slow the development of fluent sight‑reading skills. For performers with visual impairments, the findings underscore the value of interventions aimed at improving visual accessibility, such as providing enlarged notation, optimising lighting and contrast and encouraging the use of updated optical correction. More broadly, the results inform how visual accessibility should be incorporated into the design of music‑reading environments and materials, particularly in educational, rehearsal and community‑music settings.

## Data Availability

The data sets generated or analysed during this study are available from the corresponding author on reasonable request.
